# Meeting the Contraceptive Needs of Key Populations Affected by HIV in Asia: An Unfinished Agenda

**DOI:** 10.1155/2012/792649

**Published:** 2012-09-08

**Authors:** Tricia Petruney, Shanthi Noriega Minichiello, Misti McDowell, Rose Wilcher

**Affiliations:** ^1^FHI 360 Headquarters, Durham, NC 27713, USA; ^2^FHI 360 Asia and Pacific Regional Office, Bangkok 10330, Thailand; ^3^FHI 360, Dhaka 1212, Bangladesh

## Abstract

Like all women, women living with and at risk of acquiring HIV have the right to determine the number and timing of their pregnancies and to safely achieve their reproductive intentions. Yet, many women in Asia affected by HIV lack access to family planning services and experience disproportionately high rates of unintended pregnancy and abortion. Programs that have succeeded in promoting condom use and providing HIV prevention and treatment services in this region have largely missed the opportunity to address the contraceptive needs of the key populations they serve. The importance of better linkages between family planning and HIV policies and programs is now widely recognized by global health policymakers and donors. However, to date, most of the efforts to improve these linkages have been conducted in Africa. Greater attention is needed to the developing, implementing, and evaluating of integrated family planning/HIV approaches that are tailored to the political, cultural, and public health context in Asia. In this paper, we describe the use of and need for family planning among key populations affected by HIV in Asia, discuss the challenges to effectively addressing of these needs, and offer recommendations for strengthening the linkages between family planning and HIV policies and programs in the region.

## 1. Background

The HIV/AIDS epidemic in Asia is characterized as concentrated. The national adult prevalence of HIV is still under 1% in the majority of countries in the region, but exceeds 5% in many most-at-risk populations (MARPs), including men who have sex with men (MSM), injecting drug users (IDUs), sex workers, migrants, and sexual partners of these groups. Overall declines in HIV prevalence achieved in the region during the last decade are offset by pockets of high prevalence within these key populations. For example, while national HIV prevalence in India remains at a low 0.3% [1], HIV rates among some MARPs, such as female sex workers (FSWs), can be as high as 41% [2].

Despite the overall low prevalence rates, the large population sizes of many Asian nations mean that about 4.8 million people were living with HIV in the region in 2010, 1.7 million (35%) of whom were women [3]. From 1990 to 2002, the proportion of Asian women with HIV increased from 21% to 35% of all adults, but has since stabilized and has stayed below the global level of 52% [3]. While the majority of women with HIV were infected by their intimate partners, many are also at risk for or acquired HIV through sex work [3]. Substantial investments have been made in condom promotion and other HIV prevention and risk reduction programs targeting female sex workers and other women at risk for or living with HIV. Many of these programs have been successful in slowing the spread of HIV. However, attention to the other sexual and reproductive health needs of women, particularly the prevention of unintended pregnancy, has been largely absent from these programs. 

Like all women, women living with and at risk of acquiring HIV have the right to determine the number and timing of their pregnancies and to safely achieve their reproductive intentions. In addition, enabling women living with HIV to prevent unintended pregnancies is an important and cost-effective strategy for preventing mother-to-child transmission (PMTCT) of HIV [4–11]. Galvanized by public health leaders and human rights advocates, the importance of better linkages between family planning and HIV policies and programs is now widely recognized by global health policymakers and donors [12]. However, most of the advocacy, programmatic efforts, and research to improve these linkages have been conducted in Africa. Efforts to identify and address the reproductive health needs of women and men affected by HIV in Asia have been scant by comparison.

Asia differs substantially from Africa in terms of the nature of the HIV epidemic, health system organization and constraints, culture, and socioeconomic development. While some lessons for advancing family planning and HIV linkages may be drawn from Africa, the Asian context necessitates strategies tailored to the unique circumstances of the region. In this paper, we argue that greater efforts are needed to identify and implement effective strategies for meeting the contraceptive needs of women and couples affected by the concentrated HIV epidemic in Asia. Drawing on English-language published peer-reviewed literature as well as grey literature (including unpublished program reports, white papers, and conference presentations), we describe what is currently known about the use of and need for family planning among key populations affected by HIV in Asia. We then use this evidence to inform a discussion of the various challenges to effectively addressing the family planning needs of key populations in Asia, and offer recommendations for strengthening the linkages between family planning and HIV policies and programs in the region. 

## 2. Family Planning Needs among Key ****Populations in Asia

The majority of data on unintended pregnancy, family planning practices, and abortion among people affected by HIV in Asia are from female sex workers, and to a lesser degree, women and couples living with HIV. 

As a result of successful condom promotion policies and programs focused on key populations, in 2009 about half the countries in the region reported that at least 80% of female sex workers used a condom with their most recent client [3]. When used consistently and correctly, condoms are effective at preventing both sexually transmitted infections (STIs) and unintended pregnancy. Indeed, many women at risk of or living with HIV report using condoms specifically for pregnancy prevention. In Cambodia, 87.3% of the FSWs surveyed reported relying exclusively on condoms for both contraception and STI/HIV prevention [13]. In a survey of recently or currently pregnant women living with HIV in six countries in Asia, condoms were reported as the most known (88%), available (83%), used (79%), and preferred (64%) method of pregnancy prevention [14].

However, the potential for dual protection against STIs and unintended pregnancy that condoms offer is often not realized. Despite encouraging reports of condom use with clients [15–19], many FSWs do not use them consistently or correctly [14–16], and often women are forced to or will accept additional money in exchange for not using a condom [15–17]. Data from China suggests that FSWs who are also injecting drug users have particularly low rates of condom use with clients [17]. Correct and consistent condom use with regular, nonpaying partners of FSWs, such as boyfriends or husbands, is even less typical. Only 13.5% of FSWs in Bangladesh [16], between 12.7 and 45% in China [17, 20], 26.7% in Hong Kong [21], and between 11 and 50% in India [18, 19] report consistently using condoms with their regular, nonpaying sexual partners. Additional evidence from India suggests that people living with HIV who are not involved in sex work also struggle to use condoms with regular sexual partners, with one-third of men and one-quarter of women reporting inconsistent use [22]. 

Use of contraceptives other than condoms by key populations in Asia varies widely across countries. Among FSWs in India and Afghanistan who were not pregnant, infertile, or desiring pregnancy, up to 53% and 65%, respectively, were using noncondom family planning methods [19, 23]. Contraceptive use was much lower among young or HIV-positive FSWs in India [19]. Research from Cambodia also suggests use of noncondom family planning methods among FSWs is low (between 1.6 and 5.2%) [13]. Information on dual method use (condoms plus another modern family planning method) is limited, but data from India suggests it is low among FSWs [24, 25] and women and couples living with HIV [26]. 

Although not extensively documented nor uniformly calculated, existing data reveal FSWs in Asia have a higher than average unmet need for family planning compared to the general population. For example, 60% of FSWs surveyed in Bangladesh [16] had an unmet need for family planning compared to 16.8% of married women ages 15–49 [27]. In China, 47% of FSWs surveyed [20] had an unmet need for family planning, while only 2.3% of married women ages 15–49 reported an unmet need [27]. The evidence from China also suggests that FSW with unmet family planning need tend to be younger, unmarried, less likely to already have children, newer to sex work, and less educated [20]. High rates of unintended pregnancies have also been documented among key populations in Asia. One study in India found that 70% of the repeat pregnancies among women living with HIV were both unplanned and unwanted [28]. Data from a study covering six countries in Asia showed that on average 37.1% of women living with HIV reported their most recent pregnancy was unwanted (Bangladesh 33.3%, Cambodia 43.5%, India 10.3%, Indonesia 33.0%, Nepal 47.5%, and Vietnam 53.2%) [14]. 

Abortion is legal in many Asian countries, and its common use among key populations in the region provides further evidence that many experience unintended pregnancies and have an unmet need for reliable contraception. Recent or lifetime abortion rates among FSWs in several countries in Asia are often higher than the national average [13, 15, 16, 19–21, 23]. For example, compared with the 5% of all Cambodian women of reproductive age estimated to have had an abortion in 2000, more than a quarter of FSWs reported having aborted a pregnancy, despite 94% also reporting consistent use of condoms with clients [15]. Twenty-six percent of FSWs surveyed in India had experienced abortion [19], compared to a general rate of 3.1 abortions per 1000 Indian women [29]. Analyses from Vietnam also indicate higher than average rates of abortion among women living with HIV. In addition, knowledge of a positive HIV status has been associated with an increased tendency to seek an abortion [30, 31]. 

## 3. Challenges to Meeting the Contraceptive Needs of MARPs

A limited but growing body of evidence from the region suggests that greater attention is needed to meet the contraceptive needs of key populations at risk of or living with HIV. However, various political, societal, cultural, and health system challenges converge to restrict access to family planning for these women and couples. 

### 3.1. Policy Barriers and Funding Gaps

Restrictive laws and policies in several countries pose major barriers for key populations to access high-quality contraceptive services and realize their reproductive rights. For example, in India, women under the age of 18 are restricted from receiving family planning services. In Bangladesh, despite recent improvements in the policies for married women, unmarried women are officially prohibited from receiving popular and effective contraceptive methods such as injectables or implants. These types of policies present real challenges for women such as FSWs, who are often young or unmarried. 

Despite the high absolute numbers of HIV infections, HIV remains a low national health priority in most Asian countries given the low overall proportion of the population affected. As such, adequate funding for programs targeting key populations continues to be problematic. While programs reaching sex workers have been critically important in the region's HIV response, funding for these programs has been steadily declining since 2007 [3]. With shrinking funds, programs become less able to offer comprehensive services. Moreover, national disparities in the allocation of funds can prevent critical resources from reaching vulnerable populations. For example, in India, the states with high HIV prevalence also have the highest contraceptive prevalence. Although key populations in high HIV prevalence states may have a high unmet need for family planning, the majority of the government's limited resources for strengthening family planning service delivery go to other states where the contraceptive prevalence is lowest among the general population. 

Furthermore, a large proportion of HIV activities in Asia is supported through external funding. While the commitment to supporting family planning services for clients with HIV continues to grow among funders, specific guidance on how HIV/AIDS funds may be allocated for this purpose is not always clear, and program managers are often faced with difficult decisions about how to mobilize and deploy resources for integrated activities. 

### 3.2. Stigma and Discrimination

The stigmatization of key populations is distributed across the policy, program, and service delivery continuum. Stigmatization can be rooted in an overall lack of female empowerment, an individual's involvement with sex work, and/or a positive HIV status. Challenges at the policy level to ensuring access to family planning services for key populations are not limited to the aforementioned restrictive laws and funding, but also often involve pervasive and systematic discrimination by authorities across the health and legal system. Furthermore, the low political importance assigned to the sexual and reproductive health of key populations is exacerbated by the criminalization of behaviors that put key populations at risk. For example, in countries where sex work is illegal, the police may use the possession of a condom as evidence of criminalized behavior. To avoid arrest some FSWs refrain from carrying them [3], which could increase the likelihood that condoms will not be used during sexual encounters with clients. 

Female sex workers and women living with HIV are also routinely stigmatized by healthcare providers in mainstream health facilities, through refusals of service, abusive treatment, or the provision of inadequate or inappropriate care [3]. FSWs in Afghanistan have reported being less likely to visit regular health clinics due to fear of being reported to local authorities [23]. In a survey of recently pregnant women living with HIV from six Asian countries, 41.6% reported difficulty finding a gynecologist to care for them during their pregnancy due to their HIV status, and 18% were not satisfied with the confidentiality afforded to them [14]. 

This type of provider bias and discrimination is a substantial barrier to the provision of comprehensive health services, including family planning, for key populations. In a study from India, physicians and clients alike reported that the family planning counseling delivered to men and women living with HIV focused exclusively on condoms, with only minimal discussion of other contraceptive method options [26]. This data reflects a common concern among HIV program planners that providing nonbarrier family planning methods to high-risk clients will be detrimental to or even reverse the progress achieved on rates of condom use for STI/HIV prevention [13, 14]. However, evidence suggests that access to or use of noncondom contraception does not decrease condom usage among people living with or at risk of HIV [32, 33]. Additional data from several Asian countries suggest that women living with HIV are sometimes encouraged to avoid pregnancy or to undergo sterilization or abortion by their healthcare providers based on their HIV-positive status [14]. These types of limited, biased, or coercive fertility discussions not only result in unmet contraceptive need, but also are violations of women's reproductive rights. 

### 3.3. Program and Service Delivery Challenges

As a result of policy barriers and stigma, women affected by HIV often only access health services through targeted STI/HIV interventions aimed at key high-risk populations. These siloed programs, however, often miss opportunities to address clients' other sexual and reproductive health needs. For example, these programs promote and provide condoms explicitly for the prevention of STIs and HIV. They often do not directly address considerations for using condoms as a family planning method (e.g., emphasizing consistent use with regular partners), discuss the importance of dual method use, offer other more reliable contraceptive method options, or provide emergency contraception. 

Due to the narrow focus of STI and HIV services, the providers staffing these programs may not have the required training or capacity to appropriately address the family planning needs of their clients. In addition, referrals from STI/HIV sites to mainstream family planning services are currently inadequate, given the stigmatization of key populations and their general avoidance of those facilities. Even though family planning services may be geographically available through mainstream health facilities, some women at risk for or living with HIV may be reluctant to access them due to provider biases and concerns about service quality and confidentiality. 

### 3.4. Lack of Operations Research and Programmatic Guidance for Asia

Globally, the evidence base for effectively linking family planning and HIV policies and services continues to grow, with more data emerging that suggests integrating family planning and HIV services can improve health outcomes. However, little of this evidence has been generated in Asia. To date, the integrated family planning and HIV programmatic interventions implemented, evaluated, and published have been almost exclusively limited to the African setting. A 2009 systematic review of evidence for linking family planning and HIV interventions included an analysis of 16 studies, only one of which was located in Asia [34]. Moreover, that study addressed the introduction of HIV services into standard family planning programs, not the integration of family planning within HIV interventions for most-at-risk populations [34]. Thus, the evidence base of best practices for effectively meeting the contraceptive needs of key populations living with or at risk of HIV in Asia remains limited. 

A growing number of technical materials and tools are available to guide the integration of family planning and HIV programs. However, strategies tailored to a concentrated HIV epidemic are rarely included. While some elements of existing technical guidance on integrating family planning services into HIV programs are widely applicable (such as the range of contraceptive options appropriate for women and couples with HIV or the core messages to include in informed choice family planning counseling), some unique policy and programmatic considerations are required within the context of concentrated epidemics. For example, programmatic guidance for the Asia region must address issues such as the systemic stigma experienced by most-at-risk groups, particularly when accessing healthcare. This type of stigma has implications for the design of appropriate and feasible referral-based models of integrated care, where sizable investment would be needed to sensitize health care providers and foster enabling environments. 

## 4. Conclusion and Recommendations

Many women in Asia who are at risk of or living with HIV also have an unmet need for effective contraception. They experience high rates of unintended pregnancy and abortion and suffer from a lack of access to comprehensive, rights-based sexual and reproductive health services. Strategies for addressing the contraceptive needs of women and couples with HIV are emerging from research and programs in Africa [34–43]. However, the Asian context presents a scenario requiring a tailored approach for effective integration of family planning and HIV services, beyond those designed for and evaluated in an African or generalized epidemic setting. Advocates, policymakers, funders, and program planners must include considerations for concentrated epidemics within the global dialogue on strengthening the linkages between family planning and HIV services. Based on our understanding and interpretation of both global and region-specific evidence and information currently available, we offer the following recommendations for groups of key stakeholders.

### 4.1. Key Population Communities and Other Advocates

- Advocate for the immediate removal of restrictive laws and policies which bar access to the full range of contraceptive services for key populations.
- Increase the expressed demand for comprehensive sexual and reproductive health services, including family planning, from clients visiting targeted STI/HIV interventions.

### 4.2. Policymakers and Donors

- Remove legal barriers to access to comprehensive sexual and reproductive health services in existing laws, policies, and guidelines.
- Allocate adequate resources to improve linkages between targeted STI/HIV interventions and sexual and reproductive health services, including family planning, safe abortion, fertility and conception counseling, and PMTCT services.

### 4.3. Program Managers and Healthcare Providers

- Build the capacity of key population communities and networks to advocate for and raise awareness among peers of their right to and need for sexual and reproductive health services.
- Leverage the strong HIV infrastructure in the region to deliver or improve access to comprehensive sexual and reproductive services.
- Consider strategies to use postabortion care as an entry point for family planning screening, counseling, provision, or referral.
- Address biases and related stigma by sensitizing and training providers on the comprehensive reproductive rights and options for all women, including key populations.
- Promote dual method use.
- Track, monitor, and report on the impact of integrated services, including on completed referrals, method preferences and uptake, and reductions in unintended pregnancies and abortions among key populations.

### 4.4. Researchers

- Conduct operations research to identify the most effective ways of designing services to meet the contraceptive needs of key populations (including for high-risk groups such as IDUs and migrants), paying particular attention to effective dual method promotion and referral strategies.
- Communicate the contraceptive needs of key populations to decision makers, including preferences for location and delivery of services, service delivery design, and contraceptive method options.

We have a growing understanding of the unmet contraceptive needs of populations affected by HIV in the Asia region, yet a dearth of evidence about how to effectively address those needs. Investments in targeted STI/HIV interventions for key Asian populations provide an important and promising platform for reaching these groups with family planning information and services. Efforts are warranted to strengthen the linkages between family planning services and targeted STI/HIV interventions for key populations in Asia and to evaluate their impact on improving reproductive health and HIV outcomes.
